# The Deletion of US3 Gene of Pseudorabies Virus (PRV) ΔgE/TK Strain Induces Increased Immunogenicity in Mice

**DOI:** 10.3390/vaccines10101603

**Published:** 2022-09-23

**Authors:** Meng-Meng Deng, Ya-Wei Sun, Chen-Meng Ding, Xi-Ya Xu, Zi-Yi Guo, Zi-Wei Han, Chen-Zhe Lv, Jiang-Kun Qi, Yong-Tao Li, Xia Yang, Lin-Yang Yu, Lu Chen

**Affiliations:** College of Veterinary Medicine, Henan Agricultural University, Zhengzhou 450046, China

**Keywords:** pseudorabies virus, PRV ΔgE/TK/US3, US3 gene, immunogenicity

## Abstract

Re-emerging pseudorabies (PR) caused by pseudorabies virus (PRV) variant has been prevailing among immunized herds in China since 2011, indicating that commercially available PR vaccine strains couldn’t provide complete protection against novel, epidemic PRV variant. Before this study, a gE/TK-gene-deleted virus (PRV ΔgE/TK) was constructed from PRV QYY2012 variant through homologous recombination and Cre/LoxP system. Here, PRV ΔgE/TK/US3 strain was generated by deleting US3 gene based on PRV ΔgE/TK strain using the same method. The growth characteristics of PRV ΔgE/TK/US3 were analogous to that of PRV ΔgE/TK. Moreover, the deletion of US3 gene could promote apoptosis, upregulate the level of swine leukocyte antigen class I molecule (SLA-I) in vitro, and relieve inflammatory response in inoculated BALB/c mice. Subsequently, the safety and immunogenicity of PRV ΔgE/TK/US3 was evaluated as a vaccine candidate in mice. The results revealed that PRV ΔgE/TK/US3 was safe for mice, and mice vaccinated with PRV ΔgE/TK/US3 could induce a higher level of PRV-specific neutralizing antibodies and cytokines, including IFN-γ, IL-2 and IL-4, also higher level of CD8^+^ CD69^+^ Tissue-Resident Memory T cells (TRM). The results show that the deletion of US3 gene of PRV ΔgE/TK strain could induce increased immunogenicity, indicating that the PRV ΔgE/TK/US3 strain is a promising vaccine candidate for preventing and controlling of the epidemic PR in China.

## 1. Introduction

Pseudorabies (PR) is an acute disease caused by pseudorabies virus (PRV), which mainly characterizes by encephalomyelitis, reproductive disorders and reproductive failure of swine [1]. And swine is the natural and storable host of PRV, infected and invisible infected pigs are the main sources of infection. Like other Alpha-herpesviruses, PRV could establish latent infection in nervous system of the infected host, to evade the immune surveillance [2,3,4]. Latently infected piglets can be a source of reinfections when the latent viral genome is reactivated spontaneously.

Pseudorabies had been controlled after using gE-deleted PR vaccines, including Bartha-K61 strain, in combination with gE-ELISA serologic differential diagnosis in Chinese swine herds from the 1990s to 2010 [5]. Nevertheless, PR reappeared that had already been vaccinated with vaccine of swine and spread quickly since late 2011. And it was found that the re-emerging PR was caused by the PRV variant, indicating that Bartha-K61 vaccine was inefficient to provide complete protection against challenges of emerging PRV variants.

The gE and TK are main virulent genes of PRV and deletion of them not only significantly reducing invasiveness and virulence, but also maintaining good immunogenicity of PRV. Therefore, the current commercial live-attenuated PRV vaccines are produced on the basis of the deletion of gE and TK [6,7,8]. US3 is also a virulent gene of PRV, and protein encoded by US3 could help PRV to escape immune surveillance of the host, by inhibiting type I IFN expression, interfering with MHC I-mediated antigen presentation and preventing infected cells from apoptosis [9,10,11]. Therefore, PRV ΔgE/TK/US3 strain could potentially be developed into an effective commercial live-attenuated vaccine.

In this study, we constructed a live-attenuated PRV strain carrying deletions for gE, TK, and US3 using homologous recombination and Cre/LoxP system. The safety and immunogenicity of PRV ΔgE/TK/US3 strain was evaluated to determine its potential as a vaccine strain.

## 2. Materials and Methods

### 2.1. Cells and Viruses

African green monkey kidney (Vero), Swine testicle (ST) and 293T cells were cultured in Dulbecco’s modified Eagle’s medium (DMEM, Hyclone) supplemented with 10% heat-inactivated fetal bovine sera (FBS, Gibco), streptomycin (100 µg/mL), and penicillin (100 IU/mL) at 37 °C in 5% CO_2_. PRV QYY2012 strain was isolated in 2012 from a piglet with neurological symptom in Henan province of China, and PRV ΔgE/TK strain was obtained by deletion of gE and TK genes based on PRV QYY2012 strain through homologous recombination and Cre/LoxP system. All viruses were propagated in Vero cells.

### 2.2. Construction of Recombinant Plasmid and Virus

**Figure 1:**
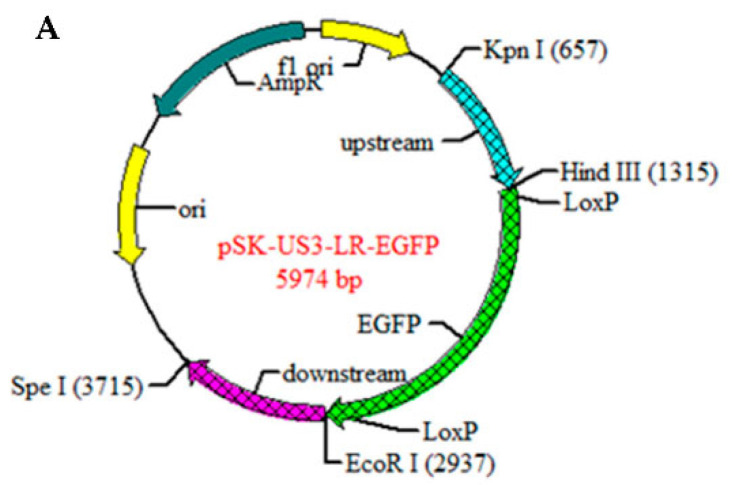

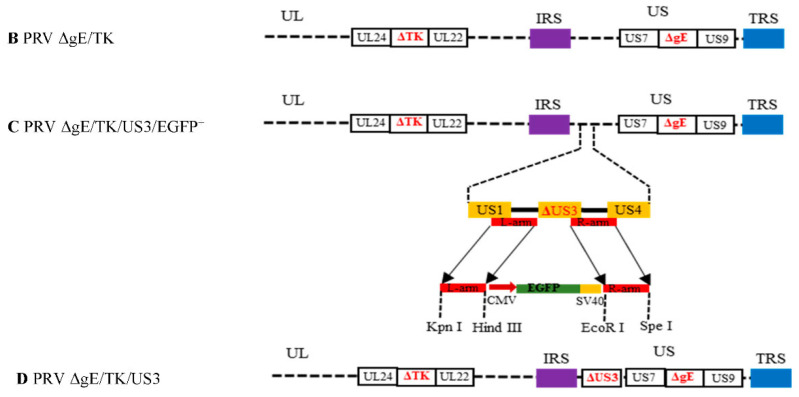
Schematic diagrams of PRV ΔgE/TK/US3. (**A**) pSK-US3-LR-EGFP recombinant plasmid. (**B**)The schematic representation of PRV ΔgE/TK genome in which partial coding regions of gE and TK genes are deleted. (**C**) The schematic representation of PRV ΔgE/TK/US3/EGFP^+^ genome in which partial coding regions of gE, TK and US3 genes are deleted and an EGFP expression cassette is inserted into the US3 deleted locus. (**D**) The schematic representation of PRV ΔgE/TK/US3 genome.

PRV QYY2012 strain genome was used as template for PCR amplification. The flanking areas of PRV US3 genes were amplified as left and right homology arms (l-arm and R-arm) using primers US3L-F/R and US3R-F/R, respectively. The L-arm contained *Kpn* Ⅰ and *Hind* Ⅲ cutting sites for directed cloning into pBlueScript SK(−) vector, the R-arm had *EcoR* Ⅰ and *Spe* Ⅰ cutting sites as well. PCR products were gel-purified and ligated to pBlueScript SK(−) vector respectively, then pSK-US3-LR recombinant vector were constructed. A signal sequence fragment, CMV-EGFP-SV40 was amplified from pEGFP plasmid by PCR using primers EGFP-F/R, which contained *Hind* Ⅲ, *EcoR* Ⅰ and LoxP sites. Enhanced green fluorescent protein (EGFP) is used as fluorescent labels to replace US3 gene during homologous recombination. The LoxP sites would be used later in the Cre/LoxP system to remove EGFP under the action of the Cre recombinant plasmid pcGlobin2-Cre. The amplified fragment was ligated into *Hind* Ⅲ- and *EcoR* Ⅰ- cleaved pSK-US3-LR vector to construct pSK-US3-LR-EGFP recombinant plasmid (Figure 1). The recombinant plasmid was confirmed by DNA sequencing. All primers used to amplify sequences are listed in Table 1.

**Figure 2:**
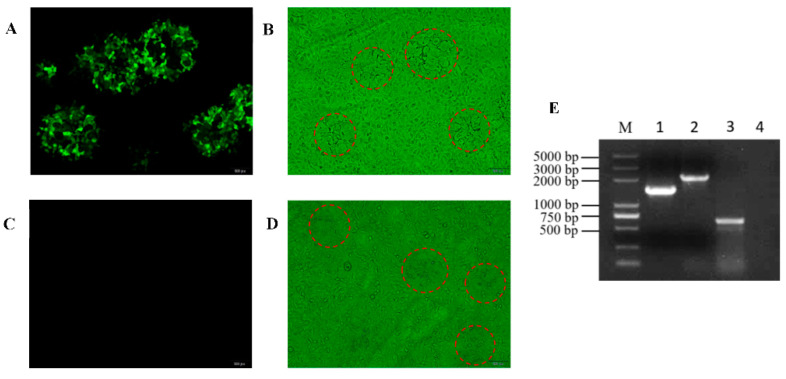
Purification and identification of PRV strains. (**A**) Plaque purification of PRV ΔgE/TK/US3/EGFP^+^ strain for fluorescence visual field. (**B**) Plaque purification of PRV ΔgE/TK/US3/EGFP^+^ strain for bright visual field, red circles represent the plaque of PRV ΔgE/TK/US3/EGFP^+^. (**C**) Plaque purification of PRV ΔgE/TK/US3 strain for fluorescence visual field. (**D**) Plaque purification of PRV ΔgE/TK/US3 strain for bright visual field, red circles represent the plaque of PRV ΔgE/TK/US3. (**E**) PCR analysis of different PRV strains with primers US3-F/R. Lane 1, PRV QYY2012; lane 2, PRV ΔgE/TK/US3/EGFP^+^; lane 3, PRV ΔgE/TK/US3; lane 4, negative control; M, DL2000 DNA Marker.

PRV ΔgE/TK strain genome DNA and pSK-US3-LR-EGFP plasmid were co-transfected into 293T cells using Lipofectamine^®^ 2000 transfection reagent (Invitrogen). When there was a significant amount of cytopathic effect (CPE) to collect the transfection mixture, and the patches with green fluorescence were selected under fluorescence microscopy. Through several rounds of purification in Vero cell to generate recombinant virus PRV ΔgE/TK/US3/EGFP^+^ (Figure 2A,B). Similarly, PRV ΔgE/TK/US3/EGFP^+^ strain genome DNA and pcGlobin2-Cre plasmid were co-transfected into 293T cells, producing recombinant virus without EGFP expression in Vero cells, hereafter referred to as PRV ΔgE/TK/US3 strain (Figure 2C,D). PRV ΔgE/TK/US3 was verified by PCR using primers US3-F/R (Table 1), and DNA sequencing to ensure that US3 gene was completely deleted (Figure 2E).

### 2.3. Growth Characteristics of PRV ΔgE/TK/US3 Strain

To analyze the growth characteristics of different PRV strains in vitro, Vero cells in 6-well plates were infected with PRV QYY2012, PRV ΔgE/TK or PRV ΔgE/TK/US3 at a multiplicity of infection (MOI) of 1 respectively. The cells were washed twice with phosphate-buffered saline (PBS) at 2 h post-infection (hpi) and supplemented with DMEM containing 2% FBS. The cell supernatants were collected at 0, 6, 12, 18, 24, 30, 36, 42, and 48 h of different post-infection time points, then virus titers were determined in 50% tissue culture infectious dose (TCID_50_) following the Reed-Muench method to establish growth curves. Three replicates were set up for each experiment.

### 2.4. Apoptosis

To assess the effects of different PRV strains on apoptosis, Vero cells in 6-well plates were infected with PRV QYY2012, PRV ΔgE/TK or PRV ΔgE/TK/US3 at MOI of 0.01. Cells were collected and filtered to single cell suspensions at 24 hpi, all experiment samples were processed according to Annexin V-FITC/PI apoptosis assay kit and examined using flow cytometry. Three replicates were set up for each experiment.

### 2.5. Swine Leukocyte Antigen Class I Molecule (SLA-I)

To detect the mRNA transcription levels of SLA-I in ST cells infected with different PRV strains, ST cells in 6-well plates were infected with PRV QYY2012, PRV ΔgE/TK or PRV ΔgE/TK/US3 at MOI of 0.1, meanwhile, pUS3 plasmid was overexpressed in ST cells as control. The cells were lysed and collected at 6 h after infection or transfection. The RNA of cell lysate was obtained according to a total RNA extraction kit, and reverse-transcribed into complementary DNA (cDNA). Using cDNA as a template, the levels of SLA-I mRNA transcription in cells were detected by real-time quantitative PCR (RT-qPCR) using primers SLA-I qF/R (Table 1). Relative expression level was ascertained after normalization against β-actin as an internal reference and calculated by 2^−∆∆Ct^. Three replicates were set up for each experiment.

### 2.6. Pro-Inflammatory Cytokines

BALB/c mice were randomly divided into 4 groups (*n* = 8/group). Mice were intramuscularly inoculated with 10^6^ TCID_50_ PRV QYY2012, PRV ΔgE/TK or PRV ΔgE/TK/US3, and injected with 100 µL of PBS serving as blank control. Mice were euthanatized and brain tissues were collected at 48 h post-injection, and RNA of brain tissues was extracted and reverse-transcribed into cDNA. The mRNA transcription levels of pro-inflammatory cytokines were detected by RT-qPCR using primers TNF-α qF/R, IL-1β qF/R or IL-6 qF/R (Table 1). Relative expression level was ascertained after normalization against GAPDH as internal reference and calculated by 2^−∆∆Ct^. Three replicates were set up for each experiment.

### 2.7. Pathogenic and Immunological Experiments in Mice

BALB/c mice were randomly divided into 4 groups (*n* = 8/group). Mice were intramuscularly injected with 10^6^ TCID_50_ PRV QYY2012, PRV ΔgE/TK or PRV ΔgE/TK/US3, and injected with 100 µL of PBS serving as blank control. The safety was evaluated by observing the clinical symptoms of each group of mice every day.

BALB/c mice were randomly divided into 4 groups (*n* = 10/group). Mice were intramuscularly immunized with 10^6^ TCID_50_ PRV ΔgE/TK/US3 or PRV ΔgE/TK, and injected with 100 µL of PBS serving as blank control. Mice were boosted with same dose at 14 days post-immunization (dpi).

Serum samples were assayed for PRV-specific NAbs by virus serum neutralizing test (NT) as previously mentioned [12,13]. Briefly, 100 μL sera in different immunization time points were collected and inactivated at 56 °C for 30 min, and incubated at 37 °C for 1 h after two-fold serial dilution using DMEM and mixture with an equal volume of 200 TCID_50_ PRV QYY2012 strain, 100 μL mixtures were inoculated into Vero cells in 96-well plates at 37 °C for 72 h and neutralizing antibody titer was calculated by the Reed-Muench method. Three replicates were set up for each experiment.

The peripheral serum of experiment mice was collected 28 days after booster immunization and used to detect the expression levels of IFN-γ, IL-2 and IL-4 by ELISA. Three replicates were set up for each experiment.

The experiment mice of trigeminal ganglion tissues were isolated at 28 days after booster immunization, and lymphocytes in trigeminal ganglion were separated by mice organ tissue lymphocyte separation kit. The lymphocytes were incubated with anti-CD8 and anti-CD69 fluorescent-labeled monoclonal antibodies for 20 min at room temperature, and the number of CD8^+^ CD69^+^ TRM cells were assessed by flow cytometry. Three replicates were set up for each experiment.

### 2.8. Statistical Analysis

The data of each experimental group were analyzed by GraphPad Prism 8.0 software (San Diego, California USA, www.graphpad.com accessed on 1 August 2022) and expressed by mean ± standard deviation (SD). For all experiments, *p* < 0.05 were considered to indicate a significant difference and *p* < 0.01 were represented an extremely significant difference.

## 3. Results

### 3.1. Generation of gE/TK/US3-Deleted Recombinant Virus

PRV ΔgE/TK genome DNA and pSK-US3-LR-EGFP plasmid were co-transfected into 293T cells, and green fluorescent plaques were selected in Vero cells and purified by plaque purification to generate PRV ΔgE/TK/US3/EGFP+ strain (Figure 2A,B). Then PRV ΔgE/TK/US3/EGFP+ genome DNA and pcGlobin2-Cre plasmid were co-transfected to generate PRV ΔgE/TK/US3 strain without EGFP expression (Figure 2C,D). PRV ΔgE/TK/US3 was verified by PCR and sequencing to confirm that 925-bp-length sequences of US3 were deleted (Figure 2E).

### 3.2. The Multiple Step Growth Curves of PRV Strains

**Figure 3:**
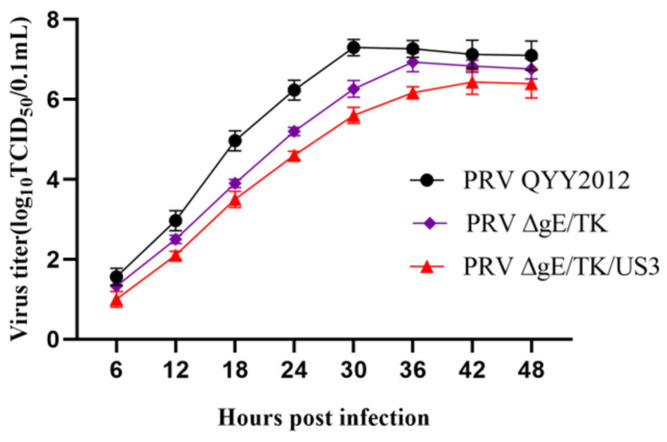
The multiple step growth curves of different PRV strains. Vero cells were inoculated with PRV QYY2012, PRV ΔgE/TK, or PRV ΔgE/TK/US3 strains. The cell culture supernatants were collected at different time points and used to calculate the TCID_50_ of each virus.

To assess growth characteristics in vitro of PRV ΔgE/TK/US3, virus replication on Vero cells were analyzed by means of the multiple step growth curves. The growth feature of PRV ΔgE/TK/US3 was virtually identical to that of PRV ΔgE/TK, and they propagated slightly slower than PRV QYY2012 strain, as shown in Figure 3.

### 3.3. PRV ΔgE/TK/US3 Strain Promotes Apoptosis

**Figure 4:**
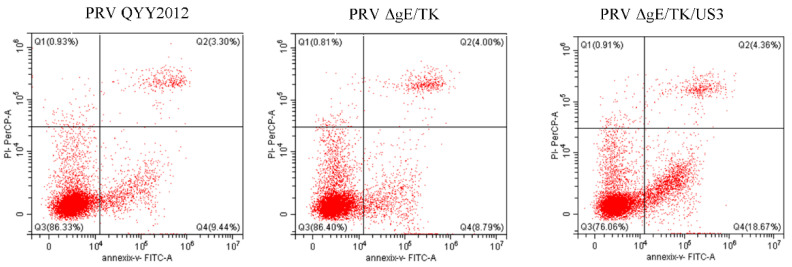
The levels of apoptosis of Vero cells infected with different PRV strains. Vero cells were inoculated with PRV QYY2012, PRV ΔgE/TK, or PRV ΔgE/TK/US3 strains. At 24 h after infection, cells were stained with Annexin V-FITC/PI and analyzed by flow cytometry.

To verify the effect of deletion of US3 gene on apoptosis, we quantified the levels of apoptosis of Vero cells infected with different PRV strains by flow cytometry. The apoptosis rates of Vero cells infected by PRV QYY2012, ΔgE/TK and ΔgE/TK/US3 strains were 12.88%, 12.81%, and 23.00%, respectively. Compared with PRV QYY2012 and ΔgE/TK strains, the percentage of apoptosis was increased by PRV ΔgE/TK/US3, and difference was extremely significant (*p* < 0.01), as shown in Figure 4. The results indicated that protein encoded by US3 could help PRV to inhibit apoptosis.

### 3.4. The Deletion of US3 Gene Upregulates the Transcriptional Level of SLA-I

**Figure 5:**
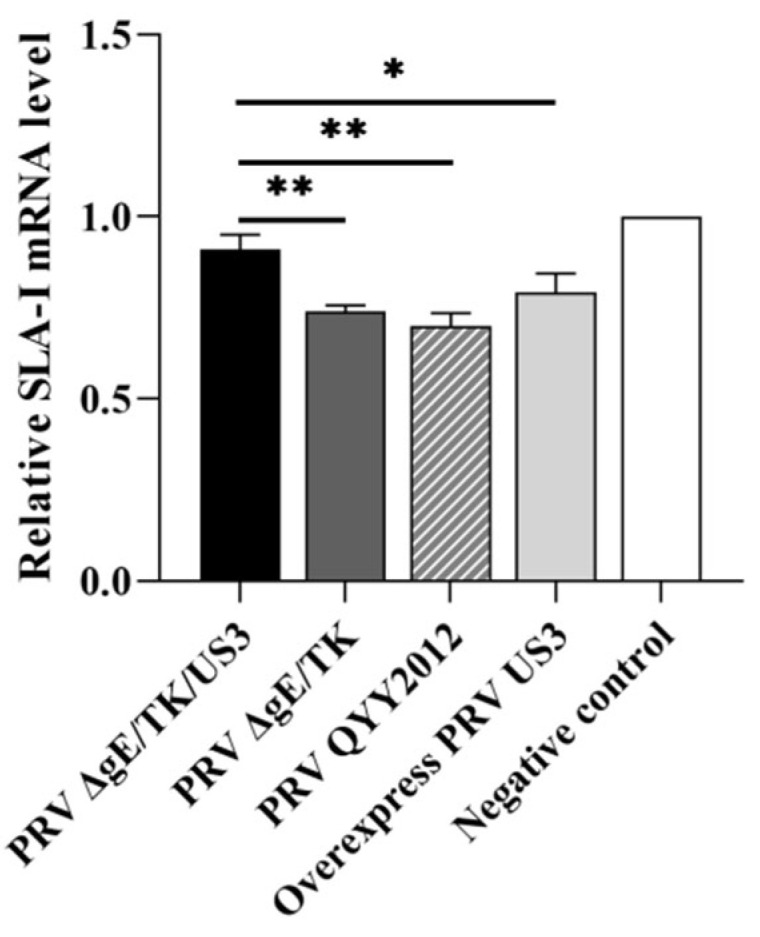
The expression level of SLA-I in ST cells infected with different PRV strains.ST cells were inoculated with PRV QYY2012, PRV ΔgE/TK or PRV ΔgE/TK/US3 strains, and pUS3 plasmid was overexpressed in ST cells. At 6 h after infection or transfection, RNA in infected ST cells was extracted and detected by RT-qPCR using primers SLA-I qF/R. Data was presented as mean ± SD.* *p* < 0.05, ** *p* < 0.01.

To investigate the effect of US3 gene on the antigen presentation, the RNA was extracted from ST cells infected with different PRV strains and used to determine the levels of SLA-I by RT-qPCR with primers SLA-I qF/R. RT-qPCR results showed that the transcriptional level of SLA-I in ST cells infected with PRV ΔgE/TK/US3 increased, and extremely significant higher than that infected with PRV QYY2012 and ΔgE/TK groups (*p* < 0.01), and significantly higher than that in pUS3 plasmid overexpression transfection group (*p* < 0.05), as shown in Figure 5. The results indicated that protein encoded by PRV US3 gene could decrease the transcriptional level of SLA-I in ST cells.

### 3.5. PRV ΔgE/TK/US3 Strain Downregulates the Levels of Pro-Inflammatory Cytokines

**Figure 6:**
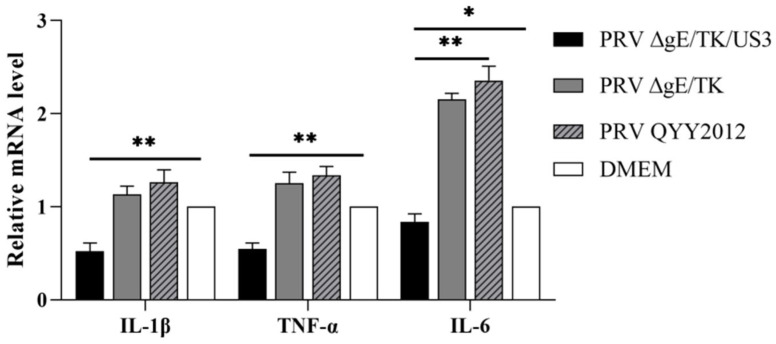
The expression level of pro-inflammatory cytokines in mice brain tissues inoculated with different PRV strains. At 48 h after inoculation, RNA in mice brain tissues was extracted and detected by RT-qPCR using primers of IL-1β, TNF-α, and IL-6. Data was presented as mean ± SD.* *p* < 0.05, ** *p* < 0.01.

To analyze the effect of US3 gene on the inflammatory response, the expression levels of pro-inflammatory cytokines were determined by RT-qPCR in brain tissues from BALB/c mice inoculated with different PRV strains. The mRNA levels of IL-1β, TNF-α, and IL-6 in brain tissue of mice infected with PRV ΔgE/TK/US3 were extremely significant lower than those in the PRV QYY2012 and ΔgE/TK groups (*p* < 0.01), as shown in Figure 6. The results suggested that deletion of US3 gene by PRV can attenuate inflammatory response in vivo.

### 3.6. The Safety and Immunogenicity of PRV ΔgE/TK/US3 in Mice

Mice inoculated with PRV ΔgE/TK, PRV ΔgE/TK/US3, and PBS all survived without clinical symptoms at 14 dpi. However, the mice inoculated with PRV QYY2012 showed typical PR symptoms and all died within 5 days after inoculation. The results indicated that PRV ΔgE/TK/US3 was safe for mice.

**Figure 7:**
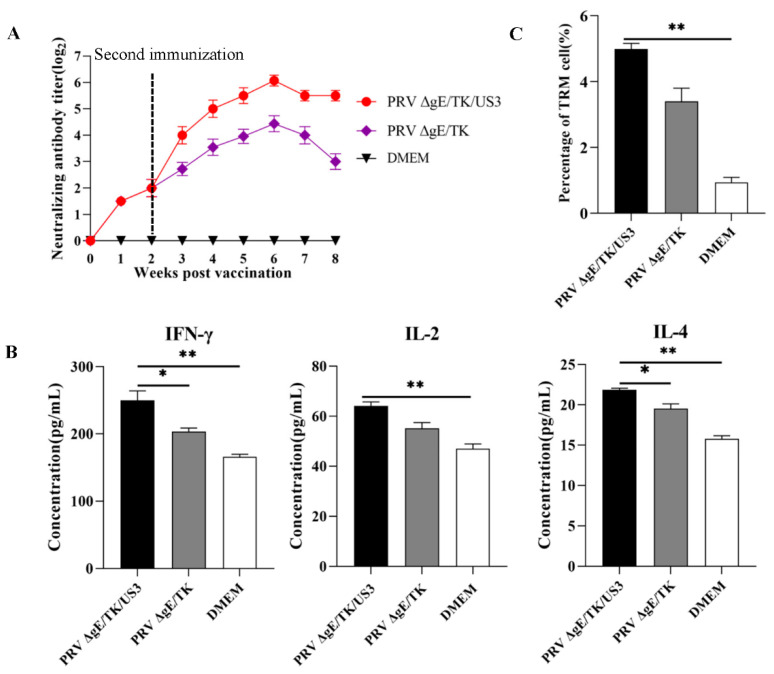
The immunogenicity of PRV ΔgE/TK/US3 in mice (**A**) Neutralizing antibody titer was examined in the serum of vaccinated mice. (**B**) The expression levels of cytokines IFN-γ, IL-2, and IL-4 in the peripheral serum of vaccinated mice were detected by ELISA. (**C**) The number of CD8^+^ CD69^+^ TRM cells in trigeminal ganglion of vaccinated mice was detected by flow cytometry. Data was presented as mean ± SD.* *p* < 0.05, ** *p* < 0.01.

The PRV-specific NAbs of serum samples were detected at every week after immunization. The results shown that mice immunized with PRV ΔgE/TK/US3 and ΔgE/TK strains produced highest levels of NAbs at 28 days after the booster immunization, and the level of NAbs in PRV ΔgE/TK/US3 group was extremely significant higher than that in PRV ΔgE/TK group (*p* < 0.01). No NAbs were induced in PBS group during experiment time, as shown in Figure 7A. Besides, the levels ofIL-2 in PRV ΔgE/TK/US3 group were extremely significant higher than in PRV ΔgE/TK group (*p* < 0.01), and the levels of IFN-γ and IL-4 were significantly higher than in PRV ΔgE/TK group (*p* < 0.05), as shown in Figure 7B. Moreover, the percentage of CD8^+^ CD69^+^ TRM cells in PRV ΔgE/TK/US3 group was extremely significant higher than in PRV ΔgE/TK and PBS groups (*p* < 0.01), as shown in Figure 7C. The results indicated that PRV ΔgE/TK/US3 had better immunogenicity than PRV ΔgE/TK.

## 4. Discussion

The Bartha-K61 vaccine had been widely used for more than 20 years in China, and PR had been well controlled. Serious PR epidemic broke out again in swine herds of China that had been vaccinated with PR vaccine since late 2011. Previous study shown that PRV variants may have resulted from gene recombination between vaccine and wild strains, which greatly increased viral virulence [14,15,16]. In addition, Bartha-K61 failed to prevent vaccinated pigs from shedding after infection and provide full protection against PRV variants [17]. Therefore, it is urgent to develop more safe and effective vaccines for controlling and eradicating virulent PRV variant.

PRV gE gene is important for virulence and gE-deleted PR vaccine has the advantages of differentiating infected from vaccinated animals along with gE ELISA diagnosis [18]. TK is another important virulence gene of PRV. The protein encoded by TK could participate in the proliferation and replication of virus in nervous cells, maintain the establishment of PRV latent infection in trigeminal ganglions of host, and play an important role in the activation of latent infection [19,20]. Therefore, deletion of TK gene could reduce viral virulence and the ability of PRV to replicate and spread on nervous cells [21]. In addition, the deletion of TK and gE genes don’t affect the immunogenicity of PRV, which would be ideal traits for vaccine candidates [22].US3 is also a virulent gene of PRV, and its product US3 protein kinase has many functions, such as inhibiting infected cells from apoptosis and regulating host immune response [23,24,25]. Previous studies showed that US3 could activate PI3-K/Akt and NF-κB pathway to protect infected cells from NK cell-mediated lysis via increased binding to CD300a receptor of the inhibitory NK cell and regulate apoptosis [26,27]. In addition, US3 could interfere type I interferon reaction by degrading Bcl-2 associated transcription factor 1 (Bclaf1), and inhibit with MHC I-mediated antigen presentation, which can promote the establishment of latent infection in PRV to some extent [28,29,30]. Therefore, US3 could also be used as a deleting target gene to investigate its immune efficacy and evaluate its potential as vaccine candidate. In this study, PRV ΔgE/TK/US3 was constructed on the basis of PRV ΔgE/TK with homologous recombination and Cre/LoxP system. Compared to PRV ΔgE/TK, PRV ΔgE/TK/US3 showed similar growth characteristics on the Vero cells. In addition, PRV ΔgE/TK/US3 could increase the level of apoptosis and SLA-I in vitro, and attenuate inflammatory response in vivo. PRV ΔgE/TK/US3 strain also could induce increased immunogenicity in mice.

During infection of cells, most viruses generate pro-apoptotic signals that activated the host immune response to limit viral replication and spread following reactivation [31]. Many viruses, including herpesviruses have evolved various strategies to suppress apoptosis of infected cells and evade cell-mediated immune response by encoding multiple anti-apoptotic genes. The US3 protein kinase encoded by herpes simplex virus type-1 (HSV-1) or PRV could block apoptosis induced by overexpression pro-apoptotic family members of Bad or Bcl-2 [32,33,34]. We also demonstrated that the function of PRV ΔgE/TK/US3 inhibiting infected cell apoptosis is decreased after deletion of US3 gene.

Viral proteins are usually processed and transported by major histocompatibility complex (MHC) class I presentation pathway and the peptide-MHC I complex are recognized by CD_8_^+^ cytotoxic T lymphocytes (CTLs) [35]. Herpesviruses could inhibit antigen presentation by MHC class I to counter CD8 T cell response and escape the immune surveillance of host [36]. HSV-1 protein ICP47 interferes with transporter associated with antigen presentation-mediated peptide loading onto MHC class I, thereby downregulating the maturation of MHC class I molecules and their presentation on the cell surface [37,38]. US2, US3, and US11 encoded by human cytomegalovirus (HCMV), a member of herpesviridae family, could interfere with MHC class I antigen presentation, thus hindering viral clearance by CTLs, specifically, US3 binds to MHC class I heavy chain complexes and components of peptide loading complex, which delay the maturation of MHC class I complexes and presentation of viral antigen on the surface of infected cells, and cooperative effect on class I down-regulation during stable expression of HCMV US2 and US3 has been established [39,40]. In present study, we confirmed that US3 gene deletion significantly increased the expression level of MHC-I (The swine origin is called swine leukocyte antigen class I molecule, SLA-I), thereby augmenting CTL recognition of infected cells in vitro.

In addition, PRV infection could increase the transcription levels of pro-inflammatory cytokines in mouse brain tissue, while US3 protein encoded by herpesvirus could resist interferon, and the mutation of US3 could attenuate inflammatory response [41,42]. Our results seemed to confirm this notion again, which US3-gene-deletion could decrease the levels of IL-1β, TNF-α, and IL-6 pro-inflammatory cytokines in brain tissue of mice infected with PRV ΔgE/TK/US3. The experimental results shown that PRV ΔgE/TK/US3 led to the loss or weakening of its function due to the deletion of US3 gene, which reduces immune escape to host, and can be recognized as foreign antigens by host’s immune system and induce a higher immune response of the body, which can be used as one of important indexes to evaluate whether PRV ΔgE/TK/US3 can be used as a candidate strain of PR vaccine.

Piglets and mice infected with PRV variants had higher mortality. The results showed high morbidity and mortality in mice infected with a gE/gI-deleted PRV mutant, but no obvious clinical symptoms in infected swine of Cong’s study [43]. Mice were considered to be more susceptible to PRV infection than swine, and could be used to evaluate the safety and immunogenicity of vaccine. In this study, we demonstrated that mice inoculated with PRV ΔgE/TK/US3 shown no clinical PR symptoms, which proved the safety of PRV ΔgE/TK/US3 as a vaccine. In addition, mice immunized with PRV ΔgE/TK/US3 that generated significantly higher levels of NAbs and cytokines, including IFN-γ, IL-2, and IL-4 than that mice immunized with PRV ΔgE/TK. Mice immunized with PRV ΔgE/TK/US3 also induced a high percentage of CD8^+^ CD69^+^ TRM cells. TRM cells have been shown to reduce recurrent infection of HSV [44,45]. The results shown that PRV ΔgE/TK/US3 as a live-attenuated vaccine strain could induce both PRV-specific cellular and humoral immune responses in mice. The immunity efficacy of PRV ΔgE/TK/US3 strain in swine will be determined in future study.

## 5. Conclusions

A gE/TK/US3-gene-deleted PRV variant was constructed by homologous recombination and Cre/LoxP system, and We demonstrated that PRV ΔgE/TK/US3 could increase the level of apoptosis and SLA-I in vitro, and attenuate inflammatory response in vivo. Then we also evaluated for its safety and immunogenicity in mice, PRV ΔgE/TK/US3 could induce positive cellular and humoral immunity, indicating that PRV ΔgE/TK/US3 has potential to be developed as an effective live-attenuated vaccine for controlling wide spreading variant strains of PRV in China. In addition, the immunity efficacy of PRV ΔgE/TK/US3 in swine and differences in immunological potency between PRV ΔgE/TK/US3 and Bartha-K61 will be determined in future study.

## Figures and Tables

**Table 1:** Primers that were used in this study.

Primers	Sequence(5′→3′)	Restriction Site	Expected Product/bp
US3L-F/R	F: CGGGGTACCGCGAGTCTGCGGGATGGT	*Kpn* Ⅰ	658
	R: CCCAAGCTTAGCGTCGAGGGCTTCTGG	*Hind* Ⅲ	
US3R-F/R	F: CCGGAATTCCTCAACAATGAAGTGGGCAAC	*EcoR* Ⅰ	778
	R: CGGACTAGTTCAGACTCCAGGCGGAAGAT	*Spe* Ⅰ	
EGFP-F/R	F: CCCAAGCTT*ATAACTTCGTATAGCATACATTATACGAAGTTAT*TTCCCATAGTAACGCCAATAG	*Hind* Ⅲ	1620
	R: CCGGAATTC*ATAACTTCGTATAATGTATGCTATACGAAGTTAT*GAAAGGACAGTGGGAGTG	*EcoR* Ⅰ	
US3-F/R	F: GGAGATGGGTCACCAAGAGG	/	1617
	R: CCGCCGTCAAAGAACCAG	/	
SLA-I qF/R	F: AAGTCAAGGAAACCGCACAG	/	113
	R: CAAGTAGCAGCCAAACATGC	/	
TNF-α qF/R	F:TGATCCGCGACGTGGAA	/	72
	R: ACCGCCTGGAGTTCTGGAA	/	
IL-1β qF/R	F: ACTCCTTAGTCCTCGGCCA	/	99
	R: CCATCAGAGGCAAGGAGGAA	/	
IL-6 qF/R	F: GAGGATACCACTCCCAACAGACC	/	141
	R: AAGTGCATCATCGTTGTTCATACA	/	
β-actin qF/R	F:TCCTGCGGCA TCCACGAAAC	/	82
	R: CCGTGTTGGCGTAGAGGTCCTTG	/	
GAPDH qF/R	F: AGGTCGGTGTGAACGGAT	/	123
	R: TGTAGACCATGTAGTTGAGG	/	

Note: bold-type letters are the sequence of restriction sites, and italics are LoxP sequence.

## References

[1] Freuling C.M., Müller T.F., Mettenleiter T.C. (2017). Vaccines against pseudorabies virus (PrV). Vet. Microbiol..

[2] Zhao H., Wang S., Liu C., Han J., Tang J., Zhou L., Ge X., Guo X., Yang H. (2018). The pUL56 of pseudorabies virus variant induces downregulation of swine leukocyte antigen class I molecules through the lysosome pathway. Virus Res..

[3] Müller T., Hahn E.C., Tottewitz F., Kramer M., Klupp B.G., Mettenleiter T.C., Freuling C. (2011). Pseudorabies virus in wild swine: A global perspective. Arch. Virol..

[4] Laval K., Enquist L.W. (2020). The Neuropathic Itch Caused by Pseudorabies Virus. Pathogens.

[5] Ao J.Q., Wang J.W., Chen X.H., Wang X.Z., Long Q.X. (2003). Expression of pseudorabies virus gE epitopes in Pichia pastoris and its utilization in an indirect PRV gE-ELISA. J. Virol. Methods.

[6] Li W., Zhuang D., Li H., Zhao M., Zhu E., Xie B., Chen J., Zhao M. (2021). Recombinant pseudorabies virus with gI/gE deletion generated by overlapping polymerase chain reaction and homologous recombination technology induces protection against the PRV variant PRV-GD2013. BMC Vet. Res..

[7] Wang J., Song Z., Ge A., Guo R., Qiao Y., Xu M., Wang Z., Liu Y., Zheng Y., Fan H. (2018). Safety and immunogenicity of an attenuated Chinese pseudorabies variant by dual deletion of TK&gE genes. BMC Vet. Res..

[8] Dong B., Zarlenga D.S., Ren X. (2014). An overview of live attenuated recombinant pseudorabies viruses for use as novel vaccines. J. Immunol. Res..

[9] Jansens R.J.J., Marmiroli S., Favoreel H.W. (2020). An Unbiased Approach to Mapping the Signaling Network of the Pseudorabies Virus US3 Protein. Pathogens.

[10] Lamote J.A.S., Glorieux S., Nauwynck H.J., Favoreel H.W. (2016). The US3 Protein of Pseudorabies Virus Drives Viral Passage across the Basement Membrane in Porcine Respiratory Mucosa Explants. J. Virol..

[11] Sehl J., Pörtner S., Klupp B.G., Granzow H., Franzke K., Teifke J.P., Mettenleiter T.C. (2020). Roles of the Different Isoforms of the Pseudorabies Virus Protein Kinase pUS3 in Nuclear Egress. J. Virol..

[12] Williams C., Wells J., Klein R., Sylvester T., Sunenshine R. (2015). Notes from the field: Outbreak of skin lesions among high school wrestlers—Arizona, 2014. MMWR Morb. Mortal. Wkly. Rep..

[13] Gravier R., Dory D., Rodriguez F., Bougeard S., Beven V., Cariolet R., Jestin A. (2007). Immune and protective abilities of ubiquitinated and non-ubiquitinated pseudorabies virus glycoproteins. Acta Virol..

[14] Delva J.L., Nauwynck H.J., Mettenleiter T.C., Favoreel H.W. (2020). The Attenuated Pseudorabies Virus Vaccine Strain Bartha K61: A Brief Review on the Knowledge Gathered During 60 Years of Research. Pathogens.

[15] Bo Z., Miao Y., Xi R., Gao X., Miao D., Chen H., Jung Y.S., Qian Y., Dai J. (2021). Emergence of a novel pathogenic recombinant virus from Bartha vaccine and variant pseudorabies virus in China. Transbound. Emerg. Dis..

[16] Zhang C., Liu Y., Chen S., Qiao Y., Guo M., Zheng Y., Xu M., Wang Z., Hou J., Wang J. (2019). A gD&gC-substituted pseudorabies virus vaccine strain provides complete clinical protection and is helpful to prevent virus shedding against challenge by a Chinese pseudorabies variant. BMC Vet. Res..

[17] Wang J., Cui X., Wang X., Wang W., Gao S., Liu X., Kai Y., Chen C. (2020). Efficacy of the Bartha-K61 vaccine and a gE(-)/gI(-)/TK(-) prototype vaccine against variant porcine pseudorabies virus (vPRV) in piglets with sublethal challenge of vPRV. Res. Vet. Sci..

[18] Cheng T.Y., Magtoto R., Henao-Díaz A. (2021). Detection of pseudorabies virus antibody in swine serum and oral fluid specimens using a recombinant gE glycoprotein dual-matrix indirect ELISA. J. Vet. Diagn. Investig..

[19] Lin J., Li Z., Feng Z., Fang Z., Chen J., Chen W., Liang W., Chen Q. (2020). Pseudorabies virus (PRV) strain with defects in gE, gC, and TK genes protects piglets against an emerging PRV variant. J. Vet. Med. Sci..

[20] Ferrari M., Gualandi G.L., Corradi A., Monaci C., Romanelli M.G., Losio M.N., Cantoni A.M., Pratelli A. (2000). The response of pigs inoculated with a thymidine kinase-negative (TK-) pseudorabies virus to challenge infection with virulent virus. Comp. Immunol. Microbiol. Infect. Dis..

[21] Tenser R.B., Ressel S.J., Fralish F.A., Jones J.C. (1983). The role of pseudorabies virus thymidine kinase expression in trigeminal ganglion infection. J. Gen. Virol..

[22] Zhao Y., Wang L.Q., Zheng H.H., Yang Y.R., Liu F., Zheng L.L., Jin Y., Chen H.Y. (2020). Construction and immunogenicity of a gE/gI/TK-deleted PRV based on porcine pseudorabies virus variant. Mol. Cell. Probes.

[23] Kadowaki N. (2021). Intratumoral cancer immunotherapy exploiting anti-viral immunity. J. Clin. Exp. Hematop..

[24] Jacob T., Van den Broeke C., Grauwet K., Baert K., Claessen C., De Pelsmaeker S., Van Waesberghe C., Favoreel H.W. (2015). Pseudorabies virus US3 leads to filamentous actin disassembly and contributes to viral genome delivery to the nucleus. Vet. Microbiol..

[25] Pomeranz L.E., Reynolds A.E., Hengartner C.J. (2005). Molecular biology of pseudorabies virus: Impact on neurovirology and veterinary medicine. Microbiol. Mol. Biol. Rev..

[26] Chang C.D., Lin P.Y., Liao M.H., Chang C.I., Hsu J.L., Yu F.L., Wu H.Y., Shih W.L. (2013). Suppression of apoptosis by pseudorabies virus Us3 protein kinase through the activation of PI3-K/Akt and NF-κB pathways. Res. Vet. Sci..

[27] Grauwet K., Vitale M., De Pelsmaeker S., Jacob T., Laval K., Moretta L., Parodi M., Parolini S., Cantoni C., Favoreel H.W. (2016). Pseudorabies Virus US3 Protein Kinase Protects Infected Cells from NK Cell-Mediated Lysis via Increased Binding of the Inhibitory NK Cell Receptor CD300a. J. Virol..

[28] Qin C., Zhang R., Lang Y., Shao A., Xu A., Feng W., Han J., Wang M., He W., Yu C. (2019). Bclaf1 critically regulates the type I interferon response and is degraded by alphaherpesvirus US3. PLoS Pathog..

[29] Geenen K., Favoreel H.W., Olsen L., Enquist L.W., Nauwynck H.J. (2005). The pseudorabies virus US3 protein kinase possesses anti-apoptotic activity that protects cells from apoptosis during infection and after treatment with sorbitol or staurosporine. Virology.

[30] Deruelle M.J., Van den Broeke C., Nauwynck H.J., Mettenleiter T.C., Favoreel H.W. (2009). Pseudorabies virus US3- and UL49.5-dependent and -independent downregulation of MHC I cell surface expression in different cell types. Virology.

[31] Carpenter D., Hsiang C., Jiang X., Osorio N., BenMohamed L., Jones C., Wechsler S.L. (2015). The herpes simplex virus type 1 (HSV-1) latency-associated transcript (LAT) protects cells against cold-shock-induced apoptosis by maintaining phosphorylation of protein kinase B (AKT). J. Neurovirol..

[32] Xie J., Zhang X., Chen L., Bi Y., Idris A., Xu S., Li X., Zhang Y., Feng R. (2021). Pseudorabies Virus US3 Protein Inhibits IFN-β Production by Interacting With IRF3 to Block Its Activation. Front. Microbiol..

[33] Zhang R., Tang J. (2021). Evasion of I Interferon-Mediated Innate Immunity by Pseudorabies Virus. Front. Microbiol..

[34] Ogg P.D., McDonell P.J., Ryckman B.J., Knudson C.M., Roller R.J. (2004). The HSV-1 Us3 protein kinase is sufficient to block apoptosis induced by overexpression of a variety of Bcl-2 family members. Virology.

[35] Imai T., Koyanagi N., Ogawa R., Shindo K., Suenaga T., Sato A., Arii J., Kato A., Kiyono H., Arase H. (2013). Us3 kinase encoded by herpes simplex virus 1 mediates downregulation of cell surface major histocompatibility complex class I and evasion of CD8+ T cells. PLoS ONE.

[36] Orr M.T., Mathis M.A., Lagunoff M., Sacks J.A., Wilson C.B. (2007). CD8 T cell control of HSV reactivation from latency is abrogated by viral inhibition of MHC class I. Cell Host Microbe.

[37] Cioni M., Mittelholzer C., Wernli M., Hirsch H.H. (2013). Comparing effects of BK virus agnoprotein and herpes simplex-1 ICP47 on MHC-I and MHC-II expression. Clin. Dev. Immunol..

[38] Raafat N., Sadowski-Cron C., Mengus C., Heberer M., Spagnoli G.C., Zajac P. (2012). Preventing vaccinia virus class-I epitopes presentation by HSV-ICP47 enhances the immunogenicity of a TAP-independent cancer vaccine epitope. Int. J. Cancer.

[39] Noriega V.M., Hesse J., Gardner T.J., Besold K., Plachter B., Tortorella D. (2012). Human cytomegalovirus US3 modulates destruction of MHC class I molecules. Mol. Immunol..

[40] Liu Z., Winkler M., Biegalke B. (2009). Human cytomegalovirus: Host immune modulation by the viral US3 gene. Int. J. Biochem. Cell Biol..

[41] Ren C.Z., Hu W.Y. (2021). Establishment of inflammatory model induced by Pseudorabies virus infection in mice. J. Vet. Sci..

[42] Wang K., Ni L., Wang S., Zheng C. (2014). Herpes simplex virus 1 protein kinase US3 hyperphosphorylates p65/RelA and dampens NF-κB activation. J. Virol..

[43] Cong X., Lei J.L., Xia S.L., Wang Y.M., Li Y., Li S., Luo Y., Sun Y., Qiu H.J. (2016). Pathogenicity and immunogenicity of a gE/gI/TK gene-deleted pseudorabies virus variant in susceptible animals. Vet. Microbiol..

[44] Roychoudhury P., Swan D.A., Duke E., Corey L., Zhu J., Davé V., Spuhler L.R., Lund J.M., Prlic M., Schiffer J.T. (2020). Tissue-resident T cell-derived cytokines eliminate herpes simplex virus-2-infected cells. J. Clin. Investig..

[45] O’Neil T.R., Hu K., Truong N.R. (2021). The Role of Tissue Resident Memory CD4 T Cells in Herpes Simplex Viral and HIV Infection. Viruses.

